# Immature Sacrococcygeal Teratoma: A Case Report and Extensive Review of the Literature

**DOI:** 10.3390/diagnostics14030246

**Published:** 2024-01-24

**Authors:** Valentin Nicolae Varlas, Eliza Maria Cloțea, Roxana Georgiana Varlas, Anca Pop, Ovidiu Peneș, Dragoș Crețoiu, Vlad Dima, Laura Bălănescu

**Affiliations:** 1 Faculty of Medicine, “Carol Davila” University of Medicine and Pharmacy, 050474 Bucharest, Romania; valentin.varlas@umfcd.ro (V.N.V.); laura.balanescu@umfcd.ro (L.B.); 2 Department of Obstetrics and Gynecology, Filantropia Clinical Hospital, 011132 Bucharest, Romania; 3 Department of Clinical Laboratory, Food Safety, “Carol Davila” University of Medicine and Pharmacy, 6 Traian Vuia Street, 020945 Bucharest, Romania; 4 Department of Intensive Care, University Clinical Hospital, “Carol Davila” University of Medicine and Pharmacy, 37 Dionisie Lupu St., 020021 Bucharest, Romania; 5 Fetal Medicine Excellence Research Center, Alessandrescu-Rusescu National Institute for Mother and Child Health, 020395 Bucharest, Romania; 6 Department of Genetics, Carol Davila University of Medicine and Pharmacy, 8 Eroii Sanitari Blvd., 050474 Bucharest, Romania; 7 Department of Neonatology, Filantropia Clinical Hospital, 011132 Bucharest, Romania; 8 Department of Pediatric Surgery, Children Emergency Hospital “Grigore Alexandrescu”, 011743 Bucharest, Romania

**Keywords:** sacrococcygeal teratoma, immature histologic type, prenatal diagnosis, therapeutic approach, recurrence, prognostic

## Abstract

Immature sacrococcygeal teratoma represents a histological form with rapid tumor growth, a risk of premature birth, an enhanced rate of complications, an increased risk of recurrence, and a higher mortality rate than the mature type. Thus, prenatal diagnosis of immature forms would significantly improve the prognosis of these cases. To this end, we performed an extensive literature review on the diagnosis, therapeutic management, and follow-up of immature teratomas. Regarding this medical conduct, we also presented our case. In conclusion, the early identification of immature sacrococcygeal teratomas with or without other associated structural abnormalities and their correct therapeutic approach are basic principles for a favorable evolution of these cases.

## 1. Introduction

Teratomas are considered rare fetal tumors, with an incidence varying between 1.3 and 2.2 in 1000 pregnancies, derived from the three germ layers [1]. Since 1975, when Santos-Ramos and Duenhoelter [2] described sonographically sacrococcygeal teratoma (SCT), the imaging technology of the prenatal diagnosis of this malformation has been continuously improved, this being highlighted by the increase in the detection rate from 50% in 2008 [3,4] to 100% in 2020 [5]. SCTs are the most common congenital tumors, arising from the multipotential cells of Hensen’s node towards the end of the second week and the beginning of the third week of gestation [6]. Teratomas usually consist of all three germ layers that form the embryo and present rapid growth; thus, their effect on the fetus will depend on their location [7]. Their development site is generally the body’s midline at any level. Teratomas can be divided in two main groups: extra-gonadal (80%) and gonadal teratomas (20%). Anatomical sites where they usually develop are, in descending order, the sacrococcygeal region (40%), pineal region (13.3%), and cervical region (13.3%) [8].

Teratomas can be divided histologically as mature when the tumor consists of well-differentiated mature tissue, immature when undifferentiated cells are present (contains neuroepithelial elements), and malignant [9].

Sacrococcygeal teratomas represent the most common tumor in fetuses and neonates [6]. Epidemiological reports on the actual incidence show a wide variability from 1:10,700 to 1:13,982 in Finland [4] and Sweden [10] to 1:27,000 in England [3], and respectively, 1:50,000 live births in Japan [11], with a mean incidence between 1:35,000 and 1:40,000 [12,13,14,15]. There is a strong predominance of tumors in female fetuses with a 4:1 ratio [16]. The increased association rate of these tumors with the female sex can be determined by the embryological differentiation of the sex at the caudal level of the mesenchymal tissue [17]. The tumor is usually diagnosed during a routine ultrasound when a complex mass in the coccyx area is identified. Doppler ultrasound, fetal echocardiography, and fetal MRI are additional diagnostic tools for a comprehensive prenatal evaluation, which guides counseling and perinatal management.

Vaginal delivery can be considered when the tumor is small. Elective cesarean delivery is generally recommended in cases of SCTs of more than 5 cm in diameter. Curative management of the tumor usually consists of tumor excision, which is performed in the first days of life [9]. The prognosis of newborns with SCT diagnosed prenatally is dependent on the histological type, the global recurrence rate varying from 2 to 35%, and the highest average value (33%) being recorded in patients with immature teratomas with a variation between 12% and 55% [18]. Unlike other studies, Rescorla et al. [19] showed that the recurrence rate is 11% in mature SCT and 4% in immature ones, whereas Dhaiban et al. [20] showed that the recurrence rate is 21.7% in mature SCT and 10% in immature ones.

**Figure 1:**
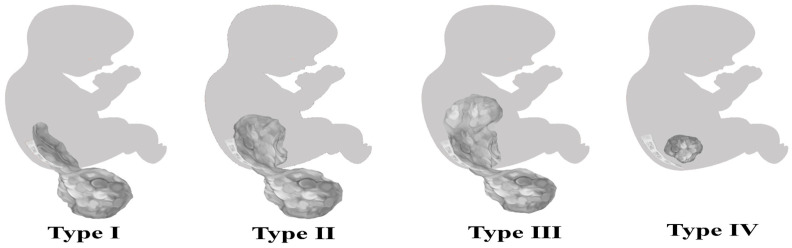
Altman classification [15]. Type I—mostly external with reduced intrapelvic extension; Type II—predominantly external with significant intrapelvic extension; Type III—predominantly intrapelvic, with visible external extension; and Type IV—intrapelvic, with possible visible external part.

Since there are few clinical studies with a large number of cases, the mortality rate of newborns with immature teratomas is not fully known, being higher than cases with mature histological type. According to the study by Yoneda et al., the mortality rate among newborns with immature teratomas was 25.8%, significantly higher than that of mature teratomas, which was 4.16% [21]. The AAPSS (American Academy of Pediatrics’ surgical section—Altman classification) (Figure 1) and Gonzalez-Crussi classification (Table 1) have an important prognostic value. The first is based on the topography of the lesion, and the second is based on histological criteria. The histological classification divides teratomas into mature with well-differentiated tissue components, immature with immature fetal tissue components in various degrees, and malignant with neoplastic germ cell content [15,22].

The unfavorable evolution of fetal SCT is determined either by heart failure secondary to an increased blood flow dependent on the intratumoral solid structure or by hemorrhage secondary to tumor rupture during vaginal delivery [23].

Monitoring during pregnancy of immature teratomas has highlighted the accelerated growth of the tumor volume, which can be associated with an increased mortality rate secondary to the risk of premature birth. The early identification of risk factors is important, the favorable evolution of SCT being dependent on the volume of the tumor, the solid component, the vascularization, the growth rate, the lack of association with other malformations, the absence of polyhydramnios, or heart failure [24]. The analysis of this information is useful to the multidisciplinary team in developing therapeutic management and counseling for future parents.

This case report and the literature review aimed to show the importance of the effective perinatal management of a complex fetal condition, including prenatal diagnosis monitoring and postnatal surgical treatment.

## 2. Case Report

We present the case of a term female newborn at 38 weeks and 5 days from a 32-year-old mother with no previous births. The pregnancy had a normal course until the 21st week, when an ultrasound scan revealed a sacrococcygeal teratoma measuring 27 × 14 × 20 mm. The first-trimester anatomy scan and double marker test indicated a low-risk profile. Non-invasive prenatal testing was performed at 22 weeks and indicated a low probability of fetal aneuploidies. The patient performed a fetal MRI at 30 weeks (Table 2).

**Table 2:** Synopsis of prenatal imagistic findings.

Type of Exam	Age	Imagistic Findings
Abdominal ultrasound	21st week	- Sacrococcygeal teratoma—27 × 20 × 14 mm, with no hypervascularity. - Amniotic fluid—normal; Doppler velocimetry—normal (Figure 2a), normal echocardiogram.
Abdominal ultrasound	29th week	- Sacrococcygeal teratoma—91.5 × 70.2 × 47.9 mm, with no hypervascularity. - Amniotic fluid—normal; Doppler velocimetry—normal (Figure 2f), normal echocardiogram.
Fetal MRI	30th week	- Expansive complex cystic mass with extra-pelvic development, with no evident intrapelvic component, measuring 78 mm in width ×74 mm anteroposterior ×54 mm craniocaudal, with a possible, solid lobular component measuring 74 × 47 × 30 mm, attached to the posterior wall. - Even though there is no evident intrapelvic component, there is a minimal mass effect on the urinary bladder and urethra. - Spinal column and thecal sac ending—normal aspect (S2); (Figure 3) Conclusion: Type I sacrococcygeal teratoma.

**Figure 2:**
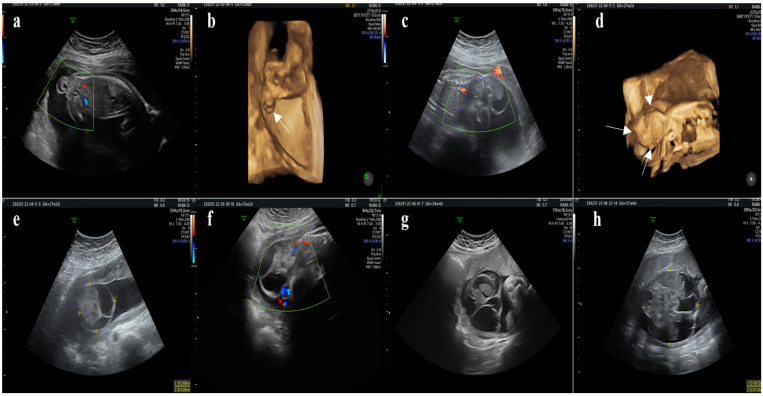
(**a**–**h**) 2D/3D ultrasound images at different gestational ages (21, 27, 31, 34, and 37 weeks) show mixed solid and cystic components originating from the coccyx without intrapelvic extension; with no hypervascularity (the white arrows show the teratoma in the 3D reconstruction).

**Figure 3:**
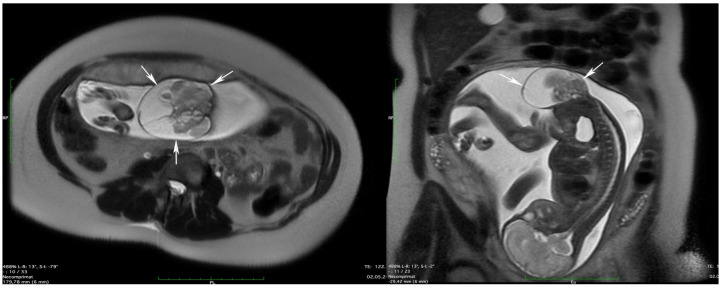
Fetal MRI. Note the type I sacrococcygeal teratoma—transversal and sagittal T2-weighted (white arrows).

A cesarean section was performed at 38 weeks and 5 days due to the large size of the teratoma. The baby was born with a birth weight of 3550 g, suitable for gestational age, and an Apgar score of 8 and 9, respectively, at 1 and 5 min. At birth, the physical examination of the newborn revealed a large sacrococcygeal tumor measuring 16 × 12 cm, covered by intact skin. Four hours after birth, the patient was admitted to the pediatric surgery department, where the following exams were performed (Table 3). The imaging evaluation did not reveal the association of any anomalies. The cardiac evaluation was normal, showing a patent foramen ovale with a left-right shunt. Serum alpha-fetoprotein was 816 ng/mL on day 2 of life.

**Figure 4:**
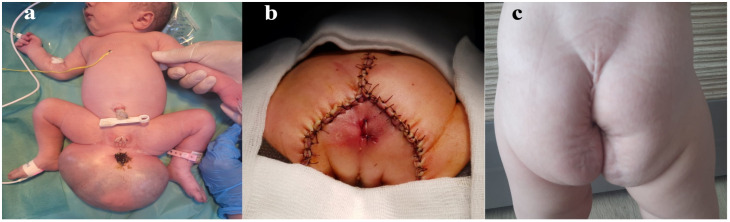
(**a**) Postpartum appearance of sacrococcygeal teratoma; (**b**) postoperative image; and (**c**) one-year follow-up.

At 2 days old, curative surgery was performed. Surgery consisted of sacrococcygeal mass excision and local drainage (Figure 4a). Intraoperative, the intimate adherence of the tumor to the rectal wall and the coccyx was observed.

The tumor was carefully dissected from the rectal wall and removed with the coccyx. The removed specimen weighed 720 g and was sent to histopathology. The surgical histopathological examination revealed a high-grade (grade 3 Gonzalez-Crussi classification), immature sacrococcygeal teratoma. The coccyx had a normal histological structure. (Table 4)

**Table 4:** Synopsis of histological findings.

**Type of Exam**	**Histological Finding**
Histopathological exam	Specimens: Sacrococcygeal mass—the tumor consists of structures derived from the three embryonic germ layers, with predominant mature neuroglial tissue, small islets resembling neuroepithelium, islets similar to the choroid plexus, islets of mature cartilaginous tissue, smooth muscle cells, islets of fatty tissue, and serous and mucinous glands; without malignant yolk sac elements. Coccyx—a fragment of coccyx—hyaline cartilage resembling a normal histological aspect. Tumoral tissue + rectal wall—a fragment of muscular and fibrous tissue, including fibers of peripheral nerves and islets with mature glands. Histopathological diagnosis: high-grade, immature sacrococcygeal teratoma (Figure 5).

**Figure 5:**
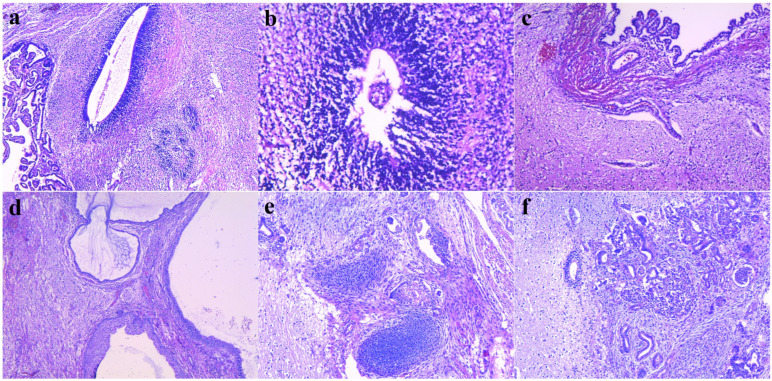
(**a**) Large tubule covered by immature neuroepithelium with small, stratified nuclei, surrounded by mature neuro-glial tissue, adjoined to papillary fronds of choroid plexus epithelium (left side), HE, 50×; (**b**) Small tubule of primitive, immature neuroepithelium, HE, 200×; (**c**) Mature neural tissue (lower part of the image) covered by thin fascicle of smooth muscle cells and simple cuboidal epithelium (upper part of the image), HE, 100×; (**d**) Disorganized mature structures formed by neural tissue (left field), cysts lined by stratified squamous epithelium (lower field), pseudostratified epithelium (right field), HE, 50×; (**e**) Immature teratoma showing small nodules of fetal cartilage, glandular structures lined by simple columnar or stratified epithelium, HE, 100×; and (**f**) immature teratoma showing neural tissue containing a small tubule of immature neuroepithelium (left side) and multiple small tubules, lined by simple cuboidal epithelium, accompanied by a glomeruloid-like structure (right side), HE, 100×.

Postoperative, the patient had a normal recovery and was discharged one week after surgery. On the 20th day of life, as a result of the immature histopathological nature of the tumor, chemotherapy treatment was initiated with Cisplatin for 5 days and Etoposide for 3 days, well tolerated clinically and biologically. Magnetic resonance imaging at 7 months showed no signs of local recurrence. One year and a half after the surgery, the follow-up showed that the patient had normal neurological and functional development and no signs of recurrence on imaging.

## 3. Review of the Literature

We used the experience of this case of immature sacrococcygeal teratoma described prenatally with favorable evolution in light of the literature review regarding the diagnosis, monitoring, perinatal management, and, respectively, the prognosis of these cases. For this purpose, a systematic electronic search was carried out using the following keywords: “immature sacrococcygeal teratoma”, “diagnosis”, “therapeutic management”, and “prognosis” in two PubMed/Medline and Web of Science databases of all time. The selection of articles required the English and French languages, clinical trials, case presentations, and case series. In total, 39 published articles were identified according to the inclusion criteria and are presented in Table 5 [3,5,10,11,13,18,19,21,22,24,25].

Following the analysis of the series of 226 cases with immature SCT, 19 G1, 29 G2, and 50 G3 were identified according to the Gonzales-Crussi classification, and 9 TI, 12 TII, 5 TIII, and 1 TIV cases according to the Altman classification; for the other cases, no data were available. After removing 26 cases with immature teratomas in which no data were available regarding their evolution, the recurrence rate was 17% (34/200 cases), and the neonatal and intrauterine deaths rate was 19.5% (39/200 cases).

## 4. Discussions

Diagnosing SCT in the first trimester of pregnancy by evaluating the spine during crown–rump length measurement or the second-trimester screening would improve the prognosis of these cases and allow early postnatal surgical intervention [54]. Thus, the early diagnosis during the first or second-trimester ultrasound allows the morphological classification of the type of SCT, the assessment of the tumor volume, the growth rate, the solid component, the vascularization, the association of other congenital defects, the presence of polyhydramnios, hydrops fetal, heart failure, or intrauterine death.

SCT can present the following ultrasound aspects: uni/multilocular cystic formation, a mixed component with cystic and solid areas, or predominantly solid. The differential diagnosis of cystic teratomas is made with the hemangioma, the neuroectodermal cyst, and the one with the mixed component with the meningomyelocele. Due to the increased morbidity and mortality of SCT, it is necessary to diagnose, if possible, from the first trimester of this condition, followed by a correct monitoring of the growth rate. Imaging cannot distinguish between mature and immature teratomas. The optimal prenatal assessment of fetal SCT requires the imaging identification of the SCT topography, the ratio of cystic and solid elements regarding the intratumoral content, the initial growth rate of the tumor volume establishes the imaging scan frequency, the degree of hypervascularization of the tumor by 3D echoangiography, the establishment RI of intratumoral arterial flows, highlighting hydrops, cardiovascular performance, and stability in utero.

The early establishment of the rapid growth rate of the tumor volume in the first two trimesters of pregnancy is necessary to identify the risk forms of SCT and to advise the parents regarding the evolution of these cases. Thus, the ultrasound identification of a large tumor with a fast growth rate, predominantly solid, hypervascular, and with blood flow at the level of intratumoral arteries with an RI that can vary between 0.60 and 0.70, represents criteria for differentiating between immature/malignant forms and the mature ones [32]. Aggravating factors are represented by the occurrence of hydrops before 30 weeks of gestation (>90% mortality) [55], cardiomegaly, ascites, pericardial effusion, absence/reversal of flow at the level of the venous duct, and heart failure [42]. Other factors that will be analyzed are polyhydramnios and the association with other congenital anomalies. This detailed analysis cannot establish the histological type of SCT; instead, it identifies the high-risk forms that are associated much more frequently, especially with immature forms and, to a lesser extent, with malignant ones.

In fetuses with increased SCT tumor volume, serial fetal echocardiography is extremely important in the careful monitoring of these patients. Cardiomegaly is assessed by measuring the cardiothoracic ratio. Circulatory changes that can occur are represented by flow disturbances in the umbilical vein and ductus venosus, dilation of the inferior vena cava, and the decrease/reversal of the diastolic flow of the umbilical artery. The combined cardiac output of fetuses with SCT can increase up to double the normal values, exceeding a flow rate of 750 mL/min/kg, being associated with cardiovascular decompensation and requiring intervention in the interests of the fetus [56].

Measuring the tumor volume of the external component is vital in planning the birth because, in the case of tumors larger than 5 cm, a cesarean section will be performed to avoid complications that may occur during birth (rupture followed by massive hemorrhages). Also, ultrasound can evaluate the intra-abdominal component of SCT, the vascularization of the tumor by power flow Doppler, and the degree of congestive heart failure by color Doppler at the level of the intratumoral entities. In the case of large and hypervascular SCT, due to the lower intratumoral vascular resistance compared to the placental one, a “vascular steal” occurs, with associated fetal anemia.

Benachi et al. made a classification based on the size and growth rate of these tumors, identifying a group of “high-risk” patients with increased mortality in the case of SCT with tumor diameter > 10 cm, important vascularization, and rapid tumor growth [57]. Therapeutic management in the case of this group of fetuses with “high-risk” SCT, with an increased mortality rate of 40–50%, can be considered the optimal time of birth between 27 and 32 weeks. As a result, prenatal identification of signs of decompensation is useful in saving these selected cases [58]. According to Benachi’s classification, our case belongs to group C (tumor diameter of 10 cm or higher, >50% cystic lesion with absent or slight vascularization, and slow growth rate), with a favorable prognosis.

Another classification of high-risk patients is based on the tumor volume, and the classification of adverse effects is based on the prenatal solid tumor volume index (STVI). Thus, Coleman et al. established that STVI > 0.09 presents an increased risk of high cardiac output state or fetal hydrops [59]. Another ratio that can be used to identify high-risk fetuses is solid tumor volume/head volume (STV/HV) [60].

Prenatal surgical procedures for SCT are not based on randomized clinical trials, and as a result, the selection of patients must be very careful, with postnatal interventions being preferred. Large sacrococcygeal tumors must be resected as soon as possible after birth due to the risk of their rupture or intratumoral bleeding secondary to the hyperdynamic circulation of teratomas with an increased vascular component [58,61].

**Figure 6:**
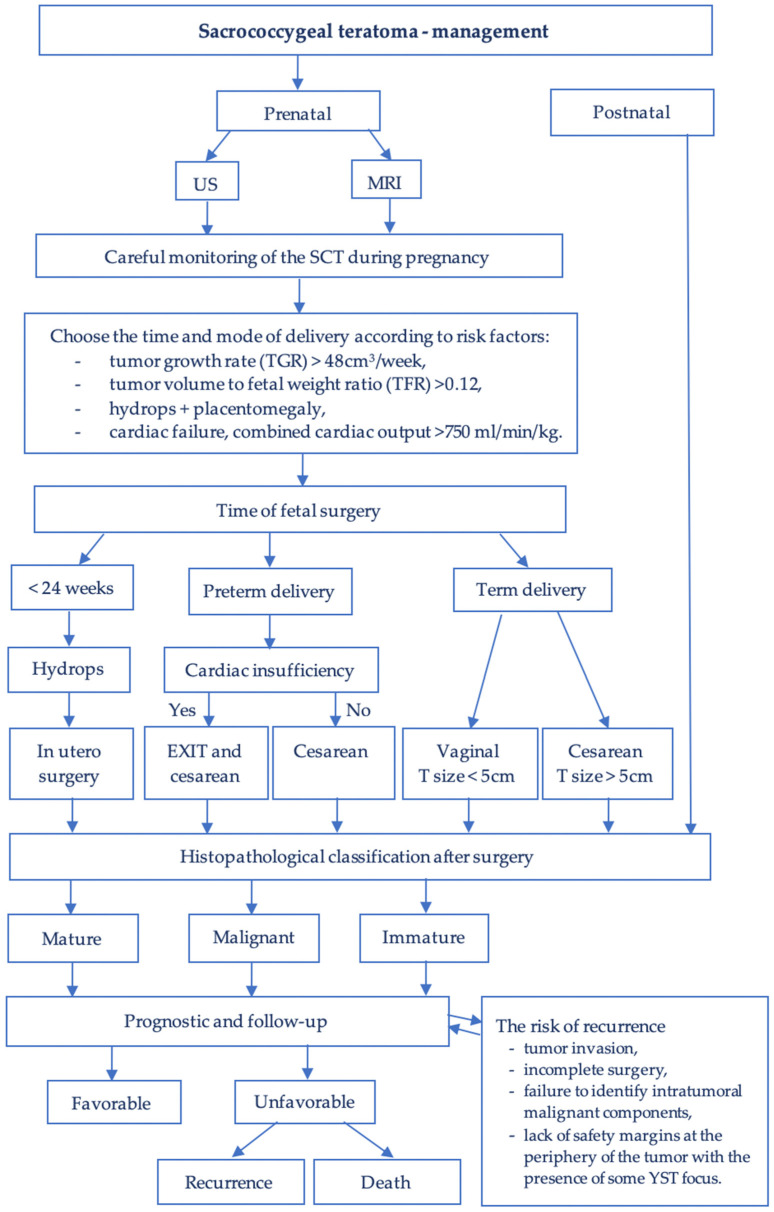
Algorithm to manage the sacrococcygeal teratoma.

However, these surgical procedures can be performed either through open or closed fetal surgery, with the main aim of reducing fetal mortality at the time of hydrops. Other possible indications for this method are the obstruction of the urinary tract by the tumor or possible dystocia in tumors with a fast growth rate. The improvement of the fetal prognosis is based on the interruption of the intratumoral vascularization, the slowing down of the growth rate of the tumor, or after its resection, on stopping or reversing the mechanisms that lead to the installation of hydrops and high-output heart failure. (Figure 6)

In addition to open fetal surgery, other methods can be used, such as percutaneously drainage, intrauterine shunt between a cystic part of the tumor and the amniotic space, ultrasound-guided percutaneous laser vessel ablation or radiofrequency tissue ablation (RFA), and alcoholic sclerosis. These interventions, which are used in the presence of fetal hydrops or circulatory insufficiency with increased flow, aim to save the fetus in utero by decoupling the tumor mass from the fetal cardiovascular system. Another study reported in solid SCT a survival rate of 57% for intratumor laser, 67% for intratumor RFA, and 20% for intratumor alcoholic sclerosis, and possible sequelae in surviving fetuses, indicating that these methods can be used in very carefully selected cases without pre-existing Doppler changes (MCA-PSV) [62]. Open or minimally invasive fetal techniques (thermal effect of laser ablation, RFA, and extratumoral spreading of sclerosing substances) can be associated with increased rates of complications (tissue destruction, hemorrhages), morbidity, and fetal mortality that varies between 30 and 60% [49].

An alternative to fetal interventions is the ex-utero intrapartum (EXIT) treatment procedure for cases with cardiac insufficiency, through which the fetus externalized from the uterus through hysterotomy is subjected to a tumor resection intervention while the mother is under general anesthesia and the fetus is placentally connected. The cesarean section is performed immediately after the baby is stabilized [63].

In the case of pregnancies with fetuses with SCT, mirror syndrome can be encountered, which is a severe form of preeclampsia, a situation in which termination of the pregnancy could be indicated [64].

Prenatally, immature teratomas show a rapid increase in size, increased risk of premature birth, a decrease in genes involved in immune processes compared to mature ones, and, respectively, increased percentages of perinatal morbidity and mortality. The treatment and monitoring of these cases must consider the risk of recurrence and the occurrence of anal and urological functional sequelae [10].

Alpha-fetoprotein (AFP) has physiologically elevated values in neonates as a result of production during embryonic development by the yolk sac and fetal liver, which reduces its utility as a tumor marker for immature teratomas in the first 4 months of neonate life [65]. Thus, although it has little utility in diagnosing malignancy in the neonatal period, it is used for clinical surveillance, having a sensitivity of 96% in diagnosing the recurrence of sacrococcygeal teratomas and malignant recurrences [66].

The risk of recurrence in the case of immature SCT can be increased in the case of tumor invasion (when the solid component is reduced), incomplete surgical intervention to remove the coccyx due to the absence of the tumor capsule, failure to identify intratumoral malignant components, as well as the lack of safety margins at the periphery of the tumor with the presence of some yolk sac tumor (YST) focus. Recurrence can occur at a variable time interval, and the histological pattern can differ from that of the initial tumor [18].

Schneider et al. showed a recurrence rate for immature forms of 33% and for mature and malignant forms of 10% and 18%, respectively [67]. The study by van Heurn et al. highlighted that in the situation of SCT with risk factors for poor outcome, all cases whose maximum diameter was >10 cm between 26 and 32 weeks of pregnancy developed heart failure and died [23]. In addition, the ascending order of the odds ratio was 1.542 for polyhydramnios, 2.311 for GA < 24 weeks, 2.39 for hypervascularization, 3.152 for the intratumoral solid component, 10.29 for cardiomegaly, and 21 for hydrops [23]. Another study shows an 8.67-fold increased risk for poor fetal prognosis in placentomegaly in fetuses with SCT [68].

Regarding the differentiation between immature SCT and malignant forms, according to Altman’s classification, most of the immature forms, according to the studies, were classified as types I/II [23], whereas the malignant forms were of type III/IV and predominantly feminine fetuses [69,70].

The average duration of follow-up, according to the study by Padillla et al. regarding the recurrence rate, is 5 years (with an interval between 5 months and 15 years) [71]; this period could not be documented in all patients.

Increased rates of recurrence were observed in incomplete resection of the tumor with margins that were not histologically free, in the impossibility of performing *en bloc* resection of the coccyx, with the lack of histological detection of malignant cellular elements in the case of large tumors, as well as in the case of which extension of the operation can cause severe functional sequelae. As for the time interval at which relapse can occur, it varies between 6 and 21 months. Despite the increased risk of recurrence, studies do not recommend postoperative chemotherapy [41]. The use of chemotherapy is controversial for immature teratomas. Thus, those with a higher histopathological grade can have foci of the yolk sac or metastasize. In the German MAKEI studies, it was evident that sacrococcygeal teratomas have a higher recurrence rate compared to other locations, and with adjuvant chemotherapy, this rate can be reduced to 9.5% [72]. The 2018 INTERIM guidelines for treating extracranial germ-cell tumors in children and adolescents recommend adjuvant chemotherapy in cases with immature teratomas, and alpha-fetoprotein above 1000 ng/mL according to the risk group [73].

Although the risk of recurrence is the main follow-up parameter, long-term functional sequelae (constipation, soiling, urinary incontinence, vicious appearance of the scar, and quality of life) represent particularly important criteria [74]. Long-term monitoring of SCT after surgical treatment is important due to the increased rate (of approximately 20%) of urological (urinary incontinence) and intestinal (constipation ± faecal soiling) sequelae [75]. As a result, counseling the family in the surgical management of these cases is decisive for a favorable prognosis.

According to the Altman classification, types III/IV of SCT and prenatal imaging evaluation that identifies intestinal or urological obstruction processes represent the selection criteria for cases that require appropriate therapy [76]. Hambraeus et al., in a controlled cohort study, identified an increased rate of sequelae (uncontrolled micturition, urinary infections, and constipation) in children with immature and large teratomas [77].

The teratoma’s malignant potential is associated with nonteratomatous elements (yolk sac tumor, embryonal carcinoma) and not with immature ones. The overall survival of SCTs in the case of local recurrence without nonteratomatous components is close to 95% and relatively good under chemotherapy in the case of the YST component [20,78].

The survival rates of SCT detected prenatally, without complications (hemorrhages, hydrops), vary between 47% and 83%. The prognosis of these cases depends on the fraction of the solid component, its vascularization, the tumor volume, and the tumor growth rate [57,59,79]. Thus, a tumor growth rate > 48 cm**^3^**/week is associated with a poor prognosis [68], whereas another study shows that a growth rate > 150 cm**^3^**/week increases the risk of perinatal mortality [80].

Rodriguez et al. analyzed the prognosis of fetuses with SCT by calculating the ratio between the volume of the tumor and the estimated weight of the fetus by ultrasound or fetal magnetic resonance, with a view to the early identification of high-risk fetuses. In pregnancy, the tumor volume-to-fetal weight ratio (TFR) cut-off was set at 0.12 before 24 weeks [81,82] and 0.11 before 32 weeks [83], with lower values being associated with a better outcome.

The molecular study of immature teratomas observed the low expression of genes involved in immune processes and significantly increased histone genes with important cellular changes [10]. In vitro and in vivo, stem-cell lines isolated from sacrococcygeal teratoma express NANOG, octamer-binding transcription factor (OCT4), neuroepithelial stem-cell protein (NESTIN), stage-specific embryonic antigen-4 (SSEA-4) and STELLA, and are negative for alpha-fetoprotein (AFP) and carcinoembryonic antigen (CEA) [84,85]. Thus, the molecular pathogenesis of these tumors is incompletely elucidated. Emerson et al. highlighted the absence of isochromosome 12p abnormalities in teratomas without a yolk sac tumor component [51].

The limitations of this study are represented by the lack of searching in as many databases as possible, which could lead to the omission of some cases, as well as the lack of reporting of relevant data in the published articles.

## 5. Conclusions

Prenatally, the difficulty of establishing by biopsy the immature histology of SCT requires the identification of high-risk cases through the imaging evaluation of unfavorable prognostic factors (predominance of the solid component, rapid tumor growth rate, and hypervascularity). This conduct allows the moment of delivery, surgical intervention, and long-term follow-up for the recurrence to be established with a view to a good perinatal prognosis.

## Figures and Tables

**Table 1:** Gonzalez-Crussi classification [22].

Grade	Percentage of Immature Tissue
0	0%
I	<10%
II	10–50%
III	>50%

**Table 3:** Synopsis of postnatal imagistic findings.

	Type of Exam	Age	Imagistic Findings
Preoperative	Abdominal ultrasound	1st day	- Liver, gallbladder, pancreas, spleen, kidneys, suprarenal glands—normal aspect; no intra/retroperitoneal free fluid. - A large extracorporeal expansive mass is emerging from the sacral region. The tumor contains cystic, solid, and fat components. Arterio-venous vascular signal in the tumor vessels. There is no clear demarcation line between the described tumor and the rectum. Small hyperechoic foci, possible microcalcifications (suspicion of teratoma).
	Transfontanelar ultrasound + chest radiography	1st day	- Normal aspect
	Radiography of lumbosacral spine	1st day	- Giant extracorporeal sacropelvic tumor measuring 157 × 127 mm. No pelvis osteolysis.
	CT of the abdomen and pelvis	2nd day	- Liver, gallbladder, pancreas, spleen, suprarenal glands, kidney, and urinary bladder of normal tridimensional aspect. No free intraperitoneal fluid. Minimal right pleural effusion. - Giant tumor mass emerging from the coccyx, mainly with extracorporeal development, spreading posteroinferior to the pelvic floor, with partial loss of the demarcation line. The presacral and intraspinal development is hard to evaluate due to the poor contrast resolution of the CT for soft tissue. The tumor measures 149 × 72 × 115 mm (T/AP/CC) and has a mixed solid-cystic structure with calcifications and a rich vascular supply. Widened ischiopubic bones due to the tumor, with no detectable osteolysis. Conclusion: Giant extrapelvic tumoral mass suggesting type I sacrococcygeal teratoma.
Postoperative	Magnetic resonance imaging (MRI)	7 months	- Surgical resection of the coccyx. No evident presacral mass. No signs of local recurrence; 1/3 of the upper segment of the rectum is visible. No regional lymphadenopathy.

**Table 5:** Overview of the immature sacrococcygeal teratoma all-time search in PubMed/Web of Science databases.

Author	Year	Age at Presentation (wks)	Cases	Grade, Type of ISCT	Diagnostic Mode	Alpha-Fetoprotein (ng/mL)	Maximum Diameter of the ISCT (cm)	Outcomes
Gonzalez- Crussi [22]	1978	24.7 (18–38) 26.6 (20–38)	2 5 3	G1 G2 G3	US	N/A	11.9 (7.5–17) 12.4 (3.8–19.5)	Recurrences—10/18 (55%)
Valdiserri [26]	1981	N/A	2 6	G2 G3	US	N/A	11.3 (2–26)	NND G3—2; death 3 years after surgery G3—1; no recurrences—4
Tapper [27]	1983	N/A	5 8 6	G1 G2 G3	US	N/A	11.6	Recurrences of G1—1; G2—1; G3—2; favorable—12; post-op death—3
Khanna [28]	1987	37	5	Not graded	US	N/A	13	N/A
Sheth [29]	1988	29 (20–35)	7	N/A	US	N/A	N/A	Recurrence at 11 m—1/7 (14.2%), elective abortion—1, IUD—2, NND—1
Johnston [30]	1988	35	1	N/A	US	191.5	20	Favorable
Havranek [31]	1992	Birth	1 1	TI TII	US	N/A	15	Recurrences—2/4 (50%), Urine leakage—1
Kirkinen [32]	1997	N/A	4	Not graded	US, MRI	N/A	12.1	NND—1, favorable—2, recurrence—1/4 (25%)
Rescorla [19]	1998	Birth	24	Not graded	US	N/A	N/A	Recurrence—1/24 (4%)
Graf [33]	1998	26.6 (26–32)	5	G3	US	N/A	N/A	NND—2, favorable—3
Uchiyama [11]	1999	30	5 (2 *)	Not graded	US	N/A	14	N/A
Marina [34]	1999	N/A	2 3	G2 G3	US	N/A	N/A	Local recurrence—1, locoregional—1, favorable—3
Herrmann [35]	2000	25 (21–31)	4	Not graded	US	N/A	13 (8.8–15)	IUD—2, NND—1, no recurrence—1
Perelli [36]	2002	N/A	3	N/A	US	N/A	N/A	Recurrence—1/3 (33%)
Huddart [37]	2003	N/A	16	N/A	US	N/A	N/A	Recurrence—2/16 (12.5%)
Iqbal [24]	2004	36	1	N/A	US	N/A	N/A	Favorable
Isaacs [38]	2004	N/A	N/A	N/A	US	N/A	N/A	N/A
Heerema-McKenney [39]	2005	26.6 (20–38)	3	G1	US	N/A	12.4 (3.8–19.5)	IUD—1; favorable—2
		24.7 (18–38)	11	G3	US	N/A	11.9 (7.5–17)	NND—1, death 3 weeks after excision—1, re-excision after 3 weeks and 14 weeks—2, intraoperative death—1, IUD—1, favorable—4
De Backer [18]	2006	N/A	5 1 1 1 1 1 1	TII TIII TII, G1 TI, G1, TIV, G1 TII, G2	US	N/A	N/A	Recurrence—2/11(18%) local Local + liver—NND Tumor spill—NND Favorable—9
Gabra [40]	2006	N/A	1	TIII	US	N/A	N/A	Neuropathic bladder, constipation, and soiling
Mann [41]	2008	32	N/A	N/A	US	N/A	N/A	NND—2, Recurrence—6/32 (18.7%), OS—93.8%,
Swamy [3]	2008	28	N/A	N/A	US	N/A	N/A	N/A
Okada [42]	2008	29	1	TIII	US	69,822	N/A	NND
Chirdan [43]	2009	9	N/A	N/A	US	N/A	N/A	N/A
Chou [44]	2011	28	1	G3	US	356.5	16	NND
Batukan [45]	2011	14	1	TII	US	-	3.3	TOP at 14 weeks
Yoneda [21]	2013	32.4 (22.7–38.8)	33	N/A	US	281.3	14	TOP—1, IUD—1, NND—8, favorable—23
Marković [46]	2013	20	1	TI	US, MRI	N/A	6.2	NND
Grammatikopoulou [47]	2013	23	1	N/A	US, MRI	N/A	10	N/A
Goto [48]	2013	27	1	TI	US, MRI	161,303	16	Death first day after surgery
Mieghem [49]	2014	26.3	1 1	TII, G3 TI	US	N/A	14	NND Mild developmental delay and mild constipation
Sarbu [50]	2016	39	1	TI, G3	US, MRI	N/A	22	Surgical wound dehiscence and sepsis, progressive active hydrocephaly secondary to chemotherapy
Emerson [51]	2016	N/A	4 2 5	G1 G2 G3	US	N/A	N/A	N/A
Bedabrata [13]	2018	N/A	1 3	G1 G2	US	N/A	N/A	N/A
Hambraeus [10]	2020	30.8 (28.4–34)	1 4 1	G1 G2 G3	US	N/A	13.8 (6.5–20)	Favorable—6
Ulm [52]	2020	23.3 (19–35)	1 3 2	TI TII TIII	US, MRI	N/A	305.2 cm^3^ (mean volume of the tumor)	TOP—1, NND—1, favorable—2, unfavorable—1, chemotherapy—1
Zheng [5]	2020	24.4 (22–29)	2 5	G2 G3	US, MRI	N/A	13.4 (9–20)	Favorable—7
Marcu [53]	2022	20 (18–22)	1 1	TI, G3 TII, G3	US	N/A	5.9 (3.3–8.5)	NND—2
Zvizdic [25]	2023	36.6 (34–38)	1 2	TI TII	US	120,516.3	12 (5–16)	Favorable—1, recurrences—2
Our case	2023	38	1	TI, G3	US, MRI	816	16	Favorable

NND—neonatal death, IUD—intrauterine death, TOP—termination of pregnancy, *—documented, wks—weeks, m—months.

## Data Availability

No new data were created or analyzed in this study. Data sharing is not applicable to this article.
